# The fabella syndrome - a rare cause of posterolateral knee pain: a review of the literature and two case reports

**DOI:** 10.1186/1471-2474-15-100

**Published:** 2014-03-26

**Authors:** Arne Driessen, Maurice Balke, Christoph Offerhaus, William James White, Sven Shafizadeh, Christoph Becher, Bertil Bouillon, Jürgen Höher

**Affiliations:** 1 Institute for Research in Operative Medicine (IFOM), University of Witten/Herdecke (Campus Cologne-Merheim), Ostmerheimerstr. 200, 51109 Cologne, Germany; 2 Department of Orthopaedic Surgery, Traumatology and Sports Traumatology, Cologne-Merheim Medical Centre (CMMC), University of Witten/Herdecke (Campus Cologne-Merheim), Ostmerheimerstr. 200, 51109 Cologne, Germany; 3 St. Vinzenz Hospital, Klinik für Unfallchirurgie, Hand- & Wiederherstellungschirurgie, Merheimer Str. 221-223, 50733 Köln-Nippes, Germany; 4 Department of Trauma and Orthopaedic Surgery, Chelsea and Westminster Hospital, NHS Foundation Trust, 369 Fulham Road, London, SW10 9NH, UK; 5 Orthopädische Klinik der Medizinischen Hochschule Hannover (MHH) im Annastift, Diakoniekrankenhaus Annastift gGmbH, Anna-von-Borries-Str. 1-7, D-30625 Hannover, Germany; 6 Department of Traumatology, Clinic for Sports Traumatology, Orthopaedic Surgery and Sports Traumatology, Cologne-Merheim Medical Centre (CMMC), University of Witten/Herdecke (Campus Cologne-Merheim), Ostmerheimerstrasse 200, 51109 Köln, Germany

**Keywords:** Fabella syndrome, Posterolateral knee pain, Fabellectomy, Sesamoid bone, Return to sports, Review of literature

## Abstract

**Background:**

The purpose of this article was to evaluate the risks and benefits of non-operative treatment versus surgical excision of a fabella causing posterolateral knee pain. We performed a systematic review of literature and also present two case reports.

Twelve publications were found in a PubMed literature review searching the word “fabella syndrome”. Non-operative treatment and surgical excision of the fabella has been described.

**Case presentation:**

Two patients presented to our outpatient clinic with persisting posterolateral knee pain. In both cases the presence of a fabella was identified, located in close proximity to the posterolateral femoral condyle. All other common causes of intra- and extra articular pathologies possibly causing the posterolateral knee pain were excluded.

Following failure to respond to physiotherapy both patients underwent arthroscopy which excluded other possible causes for posterolateral knee pain. The decision was made to undertake surgical excision of the fabella in both cases without complication.

Both patients were examined 6 month and one year after surgery with the Tegner activity score, the Visual Analogue Scale (VAS), and International Knee Documentation Committee Score (IKDC).

**Conclusion:**

Consistent posterolateral pain during exercise might indicate the presence of a fabella syndrome. Resecting the fabella can be indicated and is a minor surgical procedure with minimal risk. Despite good results in the literature posterolateral knee pain can persist and prevent return to a high level of sports. Level of evidence: IV, case reports and analysis of literature.

## Background

The fabella is a sesamoid bone in the posterolateral capsule of the human knee joint. The presence of the fabella in humans varies widely and is reported in the literature to range from 20% to 87% [1-7].

The fabella is located in the posterior aspect of the knee where lines of tensile stress intersect.

It articulates with the posterior part of the articular surface of the lateral femoral condyle and is embedded in the muscular fibres of the gastrocnemius muscle [5].

Anteriorly the fabella is bordered by the posterior capsule of the knee joint and posteriorly it is situated at the endpoint of the oblique popliteal ligament and the lateral gastrocnemius tendon. In addition the fabellofibular ligament (or lig. of Vallois) runs to its distal insertion at the fibular head.

Recent anatomic studies suggest that the presence of a fabella is higher in the Asian population [3,4,6].

Functionally, the fabella is believed to have a role similar to the patella in redirecting extension forces of the knee joint from one point to another whereas the fabella redirects forces on the flexor side [5].

Posterolateral knee pain can be associated with the presence of a fabella and this incidence may be referred to as a fabella syndrome [8-15].

It is characterized by periodic pain in the posterolateral aspect of the knee.

Pain increases with extension of the knee causing tension by pressing the fabella onto the lateral femoral condyle. Symptoms may also be present in cases in which the fabella remains non ossified as a cartilaginous structure [8].

Another major symptom caused by a fabella may be palsy of the common fibular (CF) nerve [16,17]. The CF nerve has been shown to be significantly reduced in diameter in relation to the fabella compared to proximally [1].

Ultrasound imaging may provide valuable information regarding the posterolateral structures of the knee including the presence of a fabella [18,19].

Lateral radiographs of the knee as well as MRI Imaging are able to reveal the position of a fabella in relation to the posterolateral femoral condyle.

It was the purpose of this article to firstly report about a systematic review of the current literature on the fabella syndrome and to secondly report two cases treated with this rare syndrome.

## Case presentation

### Literature search

A “pub med” research was performed using the term “fabella syndrome”. The publications were analysed for symptoms described, therapies applied, indications for surgery and clinical results reported.

### Case reports

We examined two male patients with a long history (> 12 month) of posterolateral knee pain of unknown origin who presented in our outpatient department.

Both patients underwent standard clinical examination (Lachman Test, anterior & posterior drawer Test, medial and lateral collateral ligament test, mensicus test, palpation of posterolateral aspect of the knee) and radiographic diagnostics such as ultrasound, plain radiographs (a.p. and lateral view) and MRI.

Both patients underwent previous surgery for the same symptoms which they were presenting to us.

Patient A did undergo arthroscopy with partial medial meniscectomy before presenting at our department.

**Figure 1:**
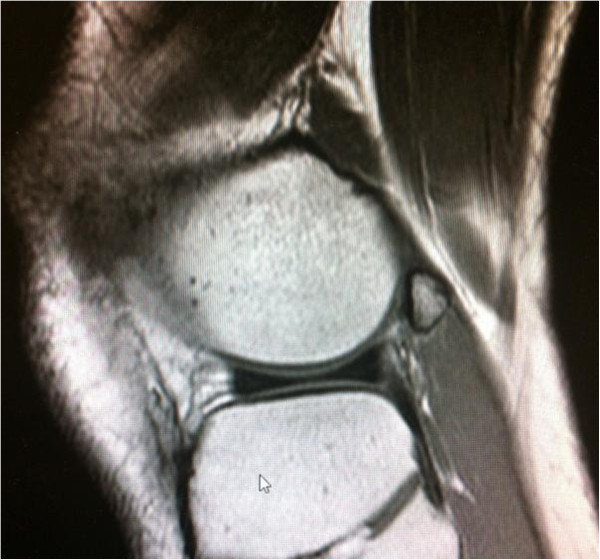
Sagittal view of lateral right knee showing the fabella in close topographical relation to the posterior lateral femoral condyle embedded in the lateral head of gastrocnemius muscle.

**Figure 2:**
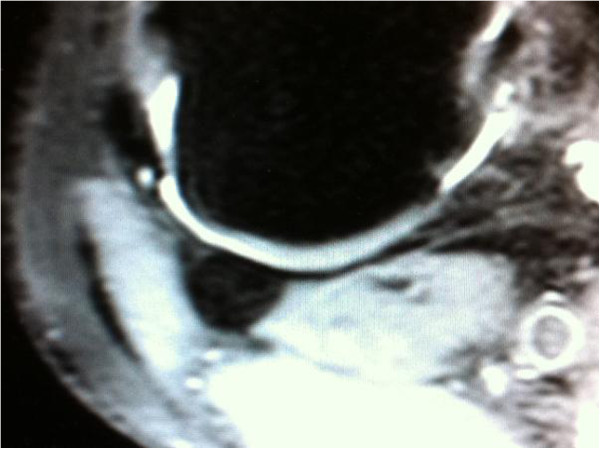
Axial view of the same knee showing the relation of the fabella to the cartilage of the posterior lateral femoral condyle.

**Figure 3:**
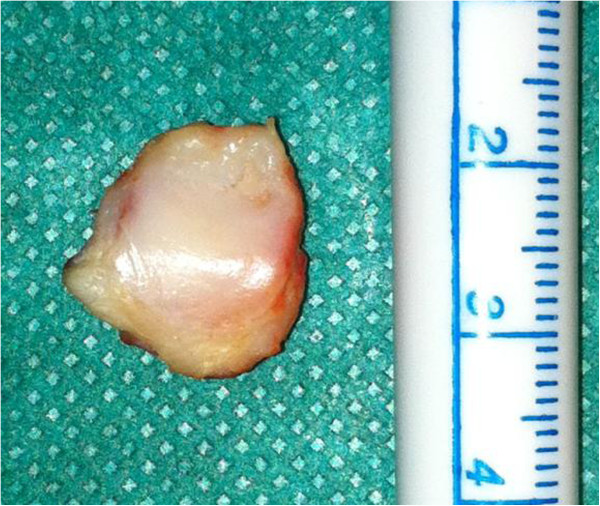
Resected fabella with cartilaginous surface.

**Figure 4:**
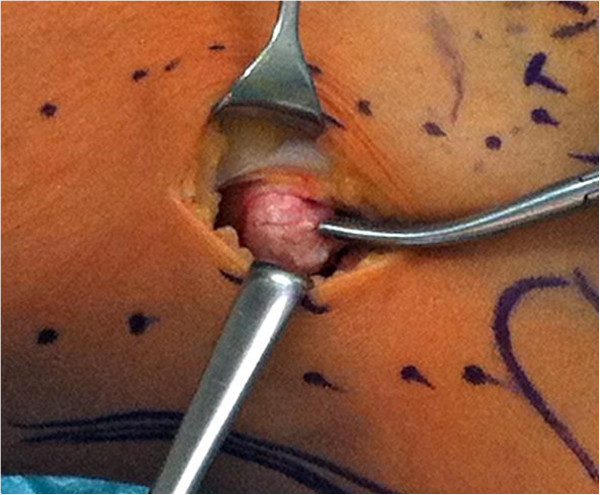
Resecting the fabella through a lateral incision between M. biceps femoris, iliotibial band and posterolateral femoral condyle.

**Figure 5:**
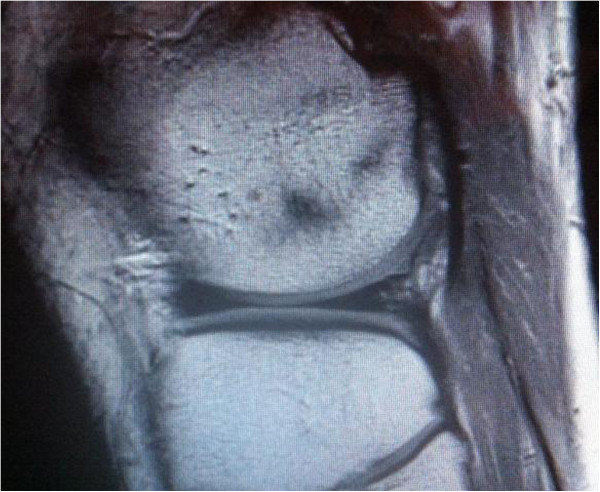
**Sagittal view after 3 month after resection of the fabella; intact posterolateral capsule & gastrocnemius muscle with little scar tissue.**

Patient B underwent bilateral knee arthroscopy before presenting to our department with complaints of persisting pain during sports in the posterolateral aspect of the right knee. Similar symptoms occurred in both sides. MRI images and pictures of the resected fabella are shown in Figure 1, 2, 3, 4 and 5.

Both patients were passionate recreational athletes and reported increasing pain whilst performing running or playing tennis with pain resolving hours after resting. Both did not report any symptoms in activities of daily living.

The activity level of both patients was determined according to the Tegner score (preoperative value of both: 3). Pain and subjective assessment of knee function were analysed using the IKDC score [20], and the Visual Analogue score (VAS). All scores were delivered preoperatively and at 6 and 12 months postoperatively.

## Results

### Literature review

The review of the literature searching the PubMed online data revealed five case reports and three studies with more than 10 patients in each paper [8-10,12,14,16,17,21].

The findings and outcome results of reported patients with the treatment of a fabella syndrome are summarized in Table 1.

**Table 1:** Details derived from six case reports found through PubMed research

	**Weiner, D.S. 1982 [**[27]**]**	**Kuur, E. 1986 [**[12]**]**	**Zipple, J.2003 [**[31]**]**	**Robertson, A. 2004 [**[22]**]**	**Dannawi, Z. 2007 [**[2]**]**	**Zenteno, B. 2010 [**[30]**]**
(n) number of patients & (morphology of fabella)	16 (9 bony & 2 cartilaginous, 5 thickened fibres of gastrocnemius muscle	1	1 (bony)	1 (bony)	2 (bony)	1 (bony)
Symptoms described in the text	Pain in extension of the knee, could be reproduced by direct pressure	3-4 year history of intermittent pain and slight swelling, painful knee extension, pain started after special intensive training	Chief complaint: left posterolateral pain, weakness and foot drop symptoms	2 month history of pain and swelling in posterolateral region of the right knee associated with a clicking sensation symptoms worse while squatting, stair ascent & descent	Case 1: sharp intermittent pain posterolateral knee, exacerbated & catching in extension case 2: pain & swelling posterolateral knee	Pain while running more than 2 km; posterolateral pain
Level of activity	Not described	Active soccer player	Lifetime habit of routine engagement in vigorous exercise 5–6 times per week	65 years old, no description about sports	58 year old women & 45 year old man, not described	High performance runner
(n) non-surgical treatment	Injections of steroid, immobilization with splinting & casting, restriction of activity, analgesics	Temporary restriction of activities, injection of steroid and anti-inflam- matory medication	1; mobilisation of the pisiform bone in the wrist	1; ultrasound guided injection of cortisone & local anaesthetic	Case 1: physical therapy for 1 year with remaining pain case 2: NSAID, no injection	Multiple conservative treatment which failed; ozone therapy, physiotherapy, ultrasound
(n) surgery	11	1	No	No	2	1
Follow up	0,5 y – 7 y	2,5 years	16 month	12 month	12 & 18 month	11 month
Symptoms & problems after procedure	12 pain free (surgical treatment); 1 minimal periodic pain, 2 periodic pain, 1 significant periodic pain (non surgical treatment); 5, injections of steroid, immobilization with splinting & casting,restriction of activity, analgesics	Work & sports without pain	None	No symptoms 12 month after intervention	Case 1: pain subsided, pain free 12 month after procedure case 2: no pain	None
Activity after surgery	No report	No report	Full 0 (0–10)	No report	No report	High, international level competitions, participant in Olympic games 2008 11 month after surgery
Nerve palsy	-		-	No report		-

Weiner et al. described the largest number of patients (n = 16) treated for pain associated with the presence of a fabella.

According to their publication five patients responded to conservative treatment. Eleven patients required surgery, all of which obtained immediate relief of symptoms with removal of the ossified fabella, cartilaginous fabella, or thickened gastrocnemius fibres [8].

Dannawi et al. presented two cases of symptomatic pain caused by the presence of a fabella. Both underwent arthroscopic resection and are reported to suffer no pain 12 month after surgery [14].

Müller also described an interesting case that undermines the theory of the fabella forming in response to appropriate stress. An 18-year-old female presented to their department having sustained a complex knee injury. Radiographs were obtained which showed no evidence of a fabella. The patient was treated non-operatively. Several years later following further presentation to the department for ongoing symptoms further imaging was obtained. These showed evidence of a fabella which had not previously been present at first review [5].

### Common fibular nerve palsy

Two reports from Japan were found describing the nerve palsy in patients as the major symptom for the fabella syndrome.

Takebe reported in 1981 about seven patients suffering from common fibular nerve palsy due to compression from fabella. Three cases were treated by surgery and four cases by conservative methods [16]. Surgery was performed more than 1,5 month after onset of symptoms. All three patients treated with surgical excision of the fabella had preoperative sensory neuropathies and one patient in addition a foot drop, which was originally thought to be the result of a lumbar disc herniation. All patients recovered after surgery with one patient regaining sensory function the day after surgery. Furthermore Takebe reports that ankle dorsiflexion steadily became stronger in the patient with the motor disfunction symptoms. Although some patients from the conservative treatment group had pathological electrophysiological examinations they were not operated. Takebe recommends conservative methods first as he observed sufficient recovery within this group, despite the underlying neuropathies proven on electrophysiological studies [16].

Matsuzaki et al. described in a book chapter nerve compression in 112 patients from constricting fascia or fabella of whom 19 were operated and followed up to an average of 4 years. Pain and dysaesthesia disappeared in all cases [17].

### Anatomical studies of the fabella complex and the relation to the common fibular nerve

Müllers description of the anatomical structures adjacent to the fabella and its function are fortified by a group of the following Japanese anatomists [5].

Kawashima et al. [4] studied the fabella and its surrounding structures in 75 knees (150 heads of the gastrocnemius muscle) from 39 Japanese cadavers. They observed 99 fabellae (66.0%) including 44 complete bony fabellae (29.3%). Of these bony fabellae, 43 (97.7%) were located in the lateral head of the gastrocnemius muscle with its surrounding structures and were positioned only on the lateral condyle of the femur. Furthermore the bony fabellae and the cartilage formed small articular cavity by cooperating with the femoral condyle. Their suggestion is that the fabella may play an important role in stabilizing the fabella complex and the femoral condyle [4].

Another anatomical study was published in 2012 investigating the role of the fabella and the relation to the common fibular nerve.

Their study describes the presence of a bony or cartilage fabella in 86,9% in the lateral head of the gastrocnemius muscle in a Chinese population [6].

They dissected 61 formalin fixed specimen to record the relationship between the common peroneal nerve & the fabella.

The anatomical relation of the fabella to the CF nerve and the head of the gastrocnemius muscle are investigated in 102 knees of 51 Japanese cadavers by Tabira et al. [1]. The presence of a fabella was observed in 70 knees (68.6%). There was a significant difference in the thickness and the width of the CF nerve adjacent to a fabella than proximal to the region. The increase in width of the CF nerve was greater for knees with fabellae weather bone or cartilage than for knees with absent fabellae. The CF nerve in the bony area directly adjacent to the fabella was noted to be thinner than in the absent fabella group. Furthermore Tabira et al. describe difference in thickness of the CF nerve between the bony and cartilage fabella.

### Miscellaneous publications

Erichsen et al. [22] described a case of a 68-year-old woman who underwent total knee replacement surgery on both knees and developed local tenderness and painful clicking in the posterolateral aspect of the knee approximately one year after arthroplasty. The lateral radiograph of the knee revealed a large fabella impinging on the prosthesis. The symptoms and signs were alleviated after a bilateral surgical excision of the fabella.

According to their experience Larson et al. [23] recommended provident excision of the fabella through the anterior approach during total knee replacement due to possible impingement.

Several other reports such as a dislocation of a fabella causing catching problems is described by Frey et al. [24] as well as posttraumatic osteoarthritic changes of the fabella causing problems could be found searching PubMed [25].

Ehara [7] describes in his recent published article in which he reviewed routine MRI of the knee of 653 cases the incidence of osteocartilaginous degeneration localized in the fabellofemoral joint in patients with osteoarthritic changes as rare (1,1%; 7 out of 623 patients mean age 64 years in Japanese population).

Literature reveals one case of a stress fracture in fabella [26]. Rare as well but also described, as accompanying more complex traumatic fracture pattern of the knee joint are fractures of the fabella [27].

Even though the fabella syndrome is seldom, one description of a case could be found, in which the posterolateral knee pain caused by an intra articular osteoid osteoma with the presence of a fabella was mistaken for a painful fabella syndrome [28].

### Case reports

Of the two patients presented in outpatients with persisting posterolateral knee pain, both showed the presence of a fabella adjacent to the posterolateral femoral condyle. Standard examination and radiographic imaging such as X-Ray and MRI could exclude other intra- and extra articular pathologies possibly causing pain.

We found the clinical examination very much beneficial to fortify the diagnosis as both patients presented with the typical symptoms such as pain while extending the knee and pain that could be reproduced by direct pressure on the fabella.

In addition and correlating with the literature both reported having pain while doing exercise but having little or no pain during all day activities [8,9,12,21].

Both patients underwent surgical excision of the fabella (patient A unilateral, patient B bilateral) through a dorsolateral incision between the biceps muscle and the iIiotibial band after having had unsatisfying reduction of pain under physiotherapy. The fabellae were resected and closure of layers was performed in standard procedure [29-31]. Other possible causes for posterolateral pain were excluded arthroscopically prior to this.

The results of the different scores are shown in Table 2 showing an improvement in activity and reduction of pain.

**Table 2:** Results of scores

	**Preop (A)**	**Preop (B)**	**6 month (A)**	**6 month (B)**	**12 month (A)**	**12 month (B)**
IKDC	66,7	59,8	75,9	82,8	85,1	88,5
VAS	10,7 (1,7/6,8/2,2)	17,5 (5,3/8,0/4,2)	22,3 (6,3/7,9/8,1)	27,5 (8,8/9,3/9,2)	28,6 (9,5/9,1/10)	30 (10/10/10)
Tegner	3	3	4	4	4	5

Patient A reported to have persistent pain six month after surgery without being back to the same level of sports. Investigations couldn’t reveal any obvious reason for these problems. But significantly important both reported to be finally pain free at 12 months.

## Discussion

Since Pancoast stated in 1909 that the presence of the fabella “has apparently little or no interest beyond the mere fact of its occasional occurrence” more than hundred years passed by [32].

Within these years science developed new methods trying to describe and understand the function of several bones of the body – also of the fabella.

In comparison to other anatomical structures, little interest has been paid to this sesamoid bone that is relatively common – and in most cases is a incidental finding on imaging.

This would support the theory of Müller that the fabella does play an important role as a structure where lines of tensile stress interact. Its presence might be explained functioning as a sesamoid structure redirecting tensile forces [5].

In his review of knee MRI of 653 patients of a Japanese population 200 (31%; age range 4–89, median 61) had a fabella. Ehara noted that the pure existence in anatomical studies is higher which might be explained with the higher age of the cadavers (age range 64 – 83, no mean age given) [7].

The increased incidence in Asian population might refer to different habits according to kneeling and squatting and therefore increased tensile forces on the flexor side of the knee. But the reason - if so - remains unclear.

As well the reports existing show an increased incidence in young active athletes without evidence for a specific sport [9,12,14,21]. The occurrence of problems in these patients might be explained with increased tensile forces on the posterior knee as well. But it remains unclear why some develop problems and others don’t.

According to our clinical experience the examination of patients with posterolateral knee pain should include palpation of the posterolateral structures as well as ultrasound examination. If specific pain can be reproduced by applying pressure on the fabella, it would be a clear indication for the fabella being the underlying cause.

Other extra- and intra-articular causes for posterolateral knee pain such as Baker’s cyst, foreign bodies, meniscal tears, localized pigmented villonodular synovitis and osteochondral fragments need to be excluded. With history of trauma ligamentous instability, tibiofibular joint mobility and fracture of fabella should be considered [10,12,31,33].

Injecting local anaesthetic and steroid for diagnostic and therapeutic purpose should be performed as first intervention.

In the majority of the few reported case particularly physically active patients are most likely to develop posterolateral knee pain caused by the fabella [9,12,21].

The problems occurring are described to be a quartet of symptoms such as “intermittent posterolateral mechanical knee pain, pain accentuation by full knee extension, localized tenderness with compression of the fabella against its corresponding condylar surface, and immediate and persistent relief with fabellectomy” [34].

This quartet introduced by Weiner in 1977 confirms the indication for surgery after already having performed it due to an eradication of pain following excision. We would therefore postulate that.

Approaching the surgical pathway should include an ultrasound-guided injection of local anaesthetic and steroids for both diagnostics and therapeutics.

Reviewing literature revealed CF nerve palsy in some patients, which could be cured by resecting the fabella [16,21].

Anatomical dissections of the posterolateral knee show a close topographical relation [16,17] between the posterior femoral condyle but as well to the CF nerve, which in fact could explain both the posterolateral pain and the nerve palsy.

Furthermore this would indicate that the most favourable therapy of the mechanical compression of other structures would be a decompression fabellectomy.

But there are reports from successful conservative treatment despite patients suffering from nerve palsy [8,16].

These patients suffering from “hyper compression” pain need to be distinguished carefully from those with mechanical locking such in arthroplasty and those with arthritic changes of the fabella causing pain.

In summary we can declare that little evidence for therapeutic option we recommend and perform exists. In some patients physiotherapy and mobilisation of the fabella seems to reduce nerve palsy and pain, others undergoing fabellectomy seem to suffer residual pain.

Although there is a tendency to believe resecting the fabella would cure the problem, it does not always do. Without knowing the exact function of the fabella and its presence it’s hard to predict outcomes.

In coincidence with younger reports, Lepoutre also reported in 1929 immediate pain relief in his 13-year-old patient after excision of the fabella, which indicated to him that posterolateral pain caused by the presence of a fabella could be eradicated with fabellectomy [35].

And despite development and research, this early observation still forms our therapeutic basis nowadays.

## Conclusion

The incidence of the fabella syndrome appears to be higher in Asian population. Presenting complaints are commonly posterolateral knee pain and CF nerve palsy.

There are limited publications about the fabella syndrome, its function and the consequence of its presence or absence is still not clear.

According to literature posterolateral knee pain caused by the presence of a fabella can be eradicated by both non-operative and surgical excision. There however were cases in which this pain persisted postoperatively.

As postoperative success is uncertain the patient should be informed of all treatment options as well as the risks and benefits. Other causes for posterolateral knee pain need to be excluded carefully before suggesting surgery.

### Ethics

The Study was carried out in accordance with the Declaration of Helsinki and within appropriate ethical framework.

### Consent

Written informed consent was obtained from the patient for publication of this Case report and any accompanying images. A copy of the written consent is available for review by the Editor of this journal.

## Competing interests

The authors declare that they have no competing interests.

## Authors’ contributions

JH and AD diagnosed the fabella syndrome and operated both patients. CO and SS were examining the patients and evaluating them clinically. MB, CO, SS, CB and BB were providing scientific support and valuable advice working on the manuscript. CB and BB furthermore helped analysing and interpreting literature and data. AD, MB and JH did perform the literature review and wrote the manuscript. WJW and MB were proof reading the manuscript, revising it critically and providing generous technical support with figures and tables. All authors have read and approved the final manuscript.

## Pre-publication history

The pre-publication history for this paper can be accessed here:

http://www.biomedcentral.com/1471-2474/15/100/prepub

## References

[B1] Tabira Y Saga T Takahashi N Watanabe K Nakamura M Yamaki K-I Influence of a fabella in the gastrocnemius muscle on the common fibular nerve in Japanese subjects Clin Anat (New York, N.Y.) 2012 26 7 893 902 doi:10.1002/ca.22153 10.1002/ca.22153 22933414

[B2] Pritchett JW The incidence of fabellae in osteoarthrosis of the knee J Bone Jt Surg 1984 66 1379 1380 6501334

[B3] Minowa T Murakami G Kura H Suzuki D Han S-H Yamashita T Does the fabella contribute to the reinforcement of the posterolateral corner of the knee by inducing the development of associated ligaments? J Orthop Sci Off J Japanese Orthop Assoc 2004 9 59 65 10.1007/s00776-003-0739-2 14767706

[B4] Kawashima T Takeishi H Yoshitomi S Ito M Sasaki H Anatomical study of the fabella, fabellar complex and its clinical implications Surg Radiol Anat SRA 2007 29 611 616 10.1007/s00276-007-0259-4 17882346

[B5] Müller W The knee - form, function and ligament reconstruction Knee - Form, Funct Ligament Reconstr 1982 New York: Springer Verlag Berlin Heidelberg 40, 96, 98, 192, 249, 252

[B6] Zeng S-X Dong X-L Dang R-S Wu G-S Wang J-F Wang D Huang H-L Guo X-D Anatomic study of fabella and its surrounding structures in a Chinese population Surg Radiol Anat SRA 2012 34 65 71 10.1007/s00276-011-0828-4 21626275

[B7] Ehara S Potentially symptomatic fabella: MR imaging review Jpn J Radiol 2013 32 1 1 5 doi:10.1007/s11604-013-0253-1 24158650 10.1007/s11604-013-0253-1

[B8] Weiner DS Macnab I The “fabella syndrome”: an update J Pediatr Orthop 1982 2 405 408 10.1097/01241398-198210000-00010 6815224

[B9] Zenteno Chávez B Morales Chaparro IF De La Torre IG Fabella syndrome in a high performance runner. Case presentation and literature review Acta Ortop Mex 2010 24 264 266 Retrieved from http://www.ncbi.nlm.nih.gov/pubmed/21305764 21305764

[B10] Robertson A Jones SCE Paes R Chakrabarty G The fabella: a forgotten source of knee pain? Knee 2004 11 243 245 10.1016/S0968-0160(03)00103-0 15194103

[B11] Legendre P Fowles JV Godin C Chondromalacia of the fabella: a case report Can J Surg J Can Chir 1986 29 102 103 3955458

[B12] Kuur E Painful fabella. A case report with review of the literature Acta Orthop Scand 1986 57 453 454 10.3109/17453678609014771 3811895

[B13] Benthien JP Brunner A A symptomatic sesamoid bone in the popliteus muscle (cyamella) Musculoskelet Surg 2010 94 141 144 10.1007/s12306-010-0083-6 21104175

[B14] Dannawi Z Khanduja V Vemulapalli KK Zammit J El-Zebdeh M Arthroscopic excision of the fabella J Knee Surg 2007 20 299 301 17993073 10.1055/s-0030-1248063

[B15] Dheer S Silverberg C Zoga AC Morrison WB A 14-year-old with lateral knee pain and locking Skeletal Radiol 2011 41 1339 1340 10.1007/s00256-011-1287-z 22072238

[B16] Takebe K Hirohata K Peroneal nerve palsy due to fabella Arch Orthop Trauma Surg 1981 99 91 95 10.1007/BF00389743 7316708

[B17] Matsuzaki A Compression syndrome of the common peroneal nerve Das Kompressionssyndrom des Nervus peronaeus communis 1996 8 73 79

[B18] Sekiya JK Jacobson JA Wojtys EM Sonographic imaging of the posterolateral structures of the knee: findings in human cadavers Arthrosc J Arthrosc Relat Surg Off Publ Arthrosc Assoc North Am Int Arthrosc Assoc 2002 18 872 881 10.1053/jars.2002.32845 12368785

[B19] Draghi F Danesino GM Coscia D Precerutti M Pagani C Overload syndromes of the knee in adolescents: Sonographic findings J Ultrasound 2008 11 151 157 10.1016/j.jus.2008.09.001 23396316 PMC3552786

[B20] Hefti F Müller W Jakob RP Stäubli HU Evaluation of knee ligament injuries with the IKDC form Knee Surg Sports Traumatol Arthrosc 1993 1 226 234 10.1007/BF01560215 8536037

[B21] Zipple JT Hammer RL Loubert PV Treatment of fabella syndrome with manual therapy: a case report J Orthop Sports Phys Ther 2003 33 33 39 10.2519/jospt.2003.33.1.33 12570284

[B22] Erichsen H Bilateral fabellar impingement after total knee replacement-a case report Acta Orthop Scand 1997 68 4 403 10.3109/17453679708996187 9310050

[B23] Larson JE Becker DA Fabellar impingement in total knee arthroplasty. A case report J Arthroplasty 1993 8 95 97 10.1016/S0883-5403(06)80114-2 8436997

[B24] Frey C Bjorkengen A Sartoris D Resnick D Knee dysfunction secondary to dislocation of the fabella Clin Orthop Relat Res 1987 Sep 223 227 3621725

[B25] Franceschi F Longo UG Ruzzini L Leonardi F Rojas M Gualdi G Denaro V Dislocation of an enlarged fabella as uncommon cause of knee pain: a case report Knee 2007 14 330 332 10.1016/j.knee.2007.03.007 17490883

[B26] Woo CC Fracture of the fabella J Manipulative Physiol Ther 1988 11 422 425 3235930

[B27] Heideman GM Baynes KE Mautz AP DuBois MS Roberts JW Fabella fracture with CT imaging: a case report Emerg Radiol 2011 18 357 361 10.1007/s10140-011-0941-z 21305331

[B28] García-Germán D Sánchez-Gutiérrez S Bueno A Carballo F López-González D Canillas F Martel J Intra-articular osteoid osteoma simulating a painful fabella syndrome Knee 2010 17 310 312 10.1016/j.knee.2010.02.008 20346681

[B29] Osti M Tschann P Künzel KH Benedetto KP Posterolateral corner of the knee: microsurgical analysis of anatomy and morphometry Orthopedics 2013 36 e1114 e1120 24025000 10.3928/01477447-20130821-11

[B30] Charalambous CP Kwaees TA Anatomical considerations in hamstring tendon harvesting for anterior cruciate ligament reconstruction Muscles Ligaments Tendons J 2012 2 253 257 23738306 PMC3666537

[B31] Kroh F Indikationen zur Eröffnung der hinteren Kapseltaschen des Kniegelenkes Arch Orthop Unfallchir 1942 42 95 115 10.1007/BF02594662

[B32] Pancoast HK Radiographic statistics of the sesamoid in the tendon of the gastrocnemius Univ Penn Med Bull 1909 22 213

[B33] Oliva F Frizziero A One step open synovectomy without adjuvant therapy for diffuse pigmented villonodular synovitis of the knee in a soccer player Muscles Ligaments Tendons J 2011 1 36 39 23738243 PMC3666461

[B34] Weiner D Macnab I Turner M The fabella syndrome Clin Orthop Relat Res 1977 Jul-Aug 213 215 598120

[B35] Lepoutre C Sesamoide douloureux (sesamoide du jumeau externe) Rev Orthop 1929 16 234 236

